# The P2X3 receptor antagonist filapixant in patients with refractory chronic cough: a randomized controlled trial

**DOI:** 10.1186/s12931-023-02384-8

**Published:** 2023-04-11

**Authors:** Christian Friedrich, Klaus Francke, Surinder S. Birring, Jan Willem K. van den Berg, Paul A. Marsden, Lorcan McGarvey, Alice M. Turner, Pascal Wielders, Isabella Gashaw, Stefan Klein, Alyn H. Morice

**Affiliations:** 1 Research and Development, Pharmaceuticals, Bayer AG, 13353 Berlin, Germany; 2 Centre for Human and Applied Physiological Sciences, School of Basic and Medical Biosciences, Faculty of Life Sciences and Medicine and King’s College Hospital, London, UK; 3 Department of Pulmonology, Isala Hospital, Zwolle, The Netherlands; 4 School of Biological Sciences, Faculty of Biology, Medicine and Health Sciences, University of Manchester and North West Lung Centre, Manchester University NHS Foundation Trust, Manchester, UK; 5 Wellcome Wolfson Institute of Experimental Medicine, School of Medicine, Dentistry and Biomedical Sciences, Queen’s University Belfast, Belfast, UK; 6 Institute of Applied Health Research, University of Birmingham, Birmingham, UK; 7 Department of Pulmonary Diseases, Catharina Hospital, Eindhoven, The Netherlands; 8 Hull York Medical School, University of Hull, Castle Hill Hospital, Cottingham, E Yorkshire UK

**Keywords:** Cough reflex sensitivity, Airway hyperreactivity, Airway hyperresponsiveness, Receptor pharmacology, P2X3 receptor antagonist, Refractory chronic cough, Taste disturbances, Proof of concept

## Abstract

**Background:**

P2X3 receptor antagonists seem to have a promising potential for treating patients with refractory chronic cough. In this double-blind, randomized, placebo-controlled study, we investigated the efficacy, safety, and tolerability of the novel selective P2X3 receptor antagonist filapixant (BAY1902607) in patients with refractory chronic cough.

**Methods:**

Following a crossover design, 23 patients with refractory chronic cough (age: 60.4 ± 9.1 years) received ascending doses of filapixant in one period (20, 80, 150, and 250 mg, twice daily, 4-days-on/3-days-off) and placebo in the other. The primary efficacy endpoint was the 24-h cough frequency on Day 4 of each dosing step. Further, subjective cough severity and health-related quality of life were assessed.

**Results:**

Filapixant at doses ≥ 80 mg significantly reduced cough frequency and severity and improved cough health-related quality of life. Reductions in 24-h cough frequency over placebo ranged from 17% (80 mg dose) to 37% (250 mg dose), reductions over baseline from 23% (80 mg) to 41% (250 mg) (placebo: 6%). Reductions in cough severity ratings on a 100-mm visual analog scale ranged from 8 mm (80 mg) to 21 mm (250 mg). No serious or severe adverse events or adverse events leading to discontinuation of treatment were reported. Taste-related adverse events occurred in 4%, 13%, 43%, and 57% of patients treated with filapixant 20, 80, 150, and 250 mg, respectively, and in 12% treated with placebo.

**Conclusions:**

Filapixant proved to be efficacious, safe, and—apart from the occurrence of taste disturbances, especially at higher dosages—well tolerated during the short therapeutic intervention.

*Clinical trial registration* EudraCT, eudract.ema.europa.eu, 2018-000129-29; ClinicalTrials.gov, NCT03535168

**Supplementary Information:**

The online version contains supplementary material available at 10.1186/s12931-023-02384-8.

## Background

P2X3 receptor antagonists seem to offer a promising potential for treating patients with refractory chronic cough (RCC) [1–5], defined as *cough that persists despite optimal treatment of presumed associated common and uncommon conditions according to best practice guidelines in an adherent* patient [6]. Although RCC and chronic cough in general are relatively common conditions [7, 8], there is currently no licensed treatment. However, there are a number of candidate P2X3 antagonists for which encouraging results from clinical studies in patients with RCC or unexplained chronic cough^1^ have been published [9–13]. The potential of P2X3 receptor antagonists in RCC was first shown in clinical trials with gefapixant, an antagonist at both the P2X3 homotrimer and the P2X2/3 heterotrimer, with similar half-maximal inhibitory concentrations (IC_50_) for the two receptors [14–17]. The highest tested dose of gefapixant significantly reduced awake cough frequency in phase 3 studies, but also led to a high frequency of taste-related adverse events (AE), mainly dysgeusia (~ 60% of patients being treated). These taste effects are attributed to off-target P2X2/3 receptor blockade, since heteromeric P2X2/3 receptors are thought to be expressed on nerve fibers innervating the tongue [18].

A highly selective P2X3 receptor antagonist could therefore have therapeutic potential in RCC, with less risk of taste disturbances attributable to P2X2/3 receptor blockade [19]. Here we present the results of a phase 1/2a study with filapixant (BAY1902607), a novel P2X3 antagonist developed by Bayer and Evotec. The drug is closely related to eliapixant (BAY1817080) [13], but has a substantially higher in vitro selectivity for P2X3 over P2X2 (data on file, Bayer). The primary objective of the study presented here was to investigate the efficacy, safety and tolerability of filapixant, including its potential to induce taste disturbances. Additionally, the multiple-dose pharmacokinetics of filapixant in RCC patients was explored.

## Methods

### Study population

This was a study in men and women with RCC. To be eligible, prospective participants had to be ≥ 18 years of age, have a body mass index ≥ 18 and ≤ 35 kg/m^2^, and have suffered, according to the investigator, for at least 1 year from chronic cough unresponsive to guideline-based treatment [20]. A score of > 40 mm on the 100-mm cough severity visual analog scale (VAS) was required at screening. Individuals with either a forced expiratory volume in 1 s of < 60% of predicted normal or a forced vital capacity of < 60%, current smokers, individuals with a history of smoking within the last 6 months before the screening visit or with > 20 pack-years in total were excluded; as were individuals with contraindications for the use of the study drug or with specific risks, conditions, or habits which could impact on the aims of the study, and individuals regularly using drugs that modulate cough. See the full list of inclusion/exclusion criteria in Additional file 1.

### Study design and treatments

**Fig. 1:**
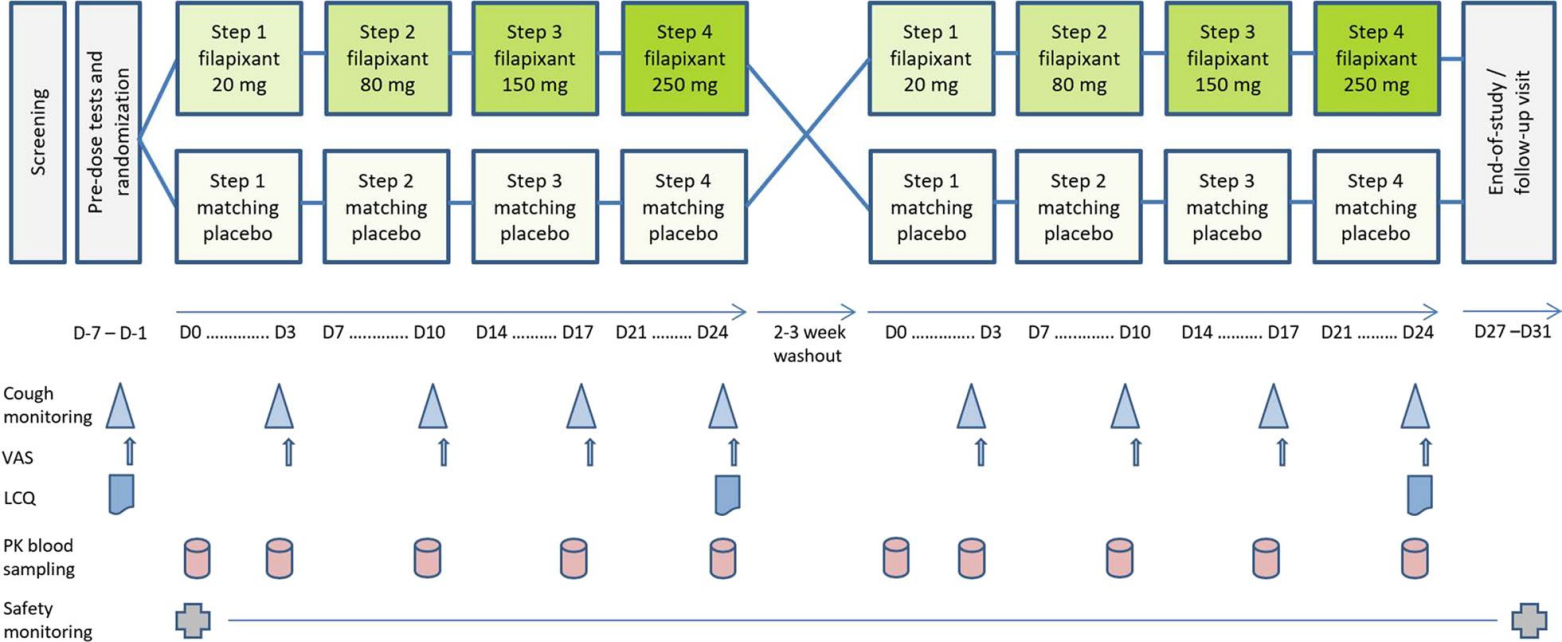
Study design and study procedures. Patients were randomized either to the treatment sequence ‘*active drug followed by placebo’* or to the sequence ‘*placebo followed by active drug’*. Doses of filapixant or placebo were administered twice daily for 4 days at each dose step. On D3, 10, 17, and 24, the patient stayed at the clinical unit until about 6 h after the morning dose and returned on the next morning. PK blood samples were taken predose and 1, 2, 4, 6, and 12 h postpose. D…, day number …; LCQ, Leicester Cough Questionnaire; PK, pharmacokinetic(s); VAS, visual analog scale

The study was designed as a double-blind, randomized, placebo-controlled, two-sequence, two-period crossover study (Fig. 1).^2^ In one period, ascending multiple oral doses of filapixant were given (20, 80, 150, and 250 mg); and matching placebo ‘doses’ were administered in the other period. The study participants were randomly assigned to one of the two possible treatment sequences using a computer-generated randomization list. At each dose step, the drug (or placebo) was taken twice daily (BID) for 4 days with a drug-free interval of 3 days between dose steps, so that cough monitoring could take place on the same day of the week at each dose step. The two treatment periods were separated by a 2- to 3-week washout period. The study was conducted under double-blind conditions, i.e., both the investigator and the participant were blinded to the treatment sequence. Active drug and placebo tablets were identical in appearance, taste and smell.

Regular use of any systemic or topical cough-modulating drugs, e.g., acetylcholine esterase inhibitors or gabapentin, within the 14 days before the first administration of study medication or during the study represented an exclusion criterion. Occasional intake of antitussives, e.g., opioids once or twice per week, was acceptable, but only > 48 h before cough monitoring to minimize the impact on study results. Stable background treatment for possible underlying cough etiologies, e.g., corticosteroids in case of asthma, was also acceptable. Based on preclinical data, no pharmacokinetic interactions with filapixant were expected in such cases.

### Procedures and variables

The primary variable for the assessment of efficacy was *24-h cough frequency* [coughs/h] measured by an ambulatory acoustic cough monitoring system (7100 VitaloJAK; Vitalograph Ltd., Maids Moreton, UK), which has previously been used in cough studies [21, 22]. Further efficacy variables were: *cough severity* as assessed by a 100-mm VAS (subjective perception of cough severity during the last 24 h; 0 = no cough; 100 mm = worst imaginable cough) and *cough health-related quality of life* as assessed by the Leicester Cough Questionnaire (LCQ; worst total score 3, best 21) [23].

Cough frequency was monitored in both treatment periods over 24 h before treatment (Day -1) and on the last treatment day of each dose step. The overall and awake mean cough frequency was determined for each 24 h period. At screening and at the end of each 24-h cough monitoring period, the participants completed the cough severity VAS; and prior to and at the end of each of the two treatment periods, they completed the LCQ.

Primary variables for the assessment of safety and tolerability were the frequency and severity of treatment-emergent AEs (observed AEs, mentioned upon open questioning, and spontaneously reported AEs). Further safety assessments included standard clinical laboratory tests, vital signs, electrocardiograms, and physical examinations.

Blood samples to determine filapixant concentrations in plasma were taken at the time points indicated in Fig. 1. The concentrations of filapixant in plasma were determined with a validated analytical method; details in Additional file 2.

### Statistical and sample size considerations

Statistical analyses were carried out using the software SAS v9.4 (SAS Institute, Cary, North Carolina, USA).

For cough count data, a lognormal distribution was assumed, i.e., the data were log-transformed and a mixed model was applied, using time point, treatment sequence, and their interaction as fixed effects. Dependence within subjects was modeled using normally distributed subject effects; between subjects, independence was assumed. This model was analyzed using a Bayesian approach with non-informative prior. A similar method was used for VAS data. The LCQ total and domain scores were analyzed by a hierarchical model, assuming normally distributed errors, using the above approach. Responder analyses were run for 24-h cough count and VAS data. The cut-off points for a positive response were: (1) > 30% reduction in cough frequency, (2) > 50% reduction in cough frequency, and (3) > 30 mm reduction in VAS cough severity (from baseline).

Statistical methods were chosen to facilitate the internal proof-of-concept decision. Due to the Bayesian statistical approach that was used throughout the study, 90% credible intervals were calculated. *P* values were determined using a one-sided hypothesis. All analyses were *exploratory*; therefore, no multiplicity adjustments were made. Placebo data from different dose steps were pooled. Baseline values were defined as the last non-missing value before the start of treatment. All study subjects who received at least one dose of the study medication were included in the safety/tolerability analysis set and all subjects who completed the study without validity findings were included in the efficacy analysis set (per-protocol analysis set).

The required sample size was estimated by simulations with SAS v9.4. Based on cough frequency data reported for another P2X3 receptor antagonist [9], a within-subject coefficient of variation of 50% (worst-case scenario) was assumed and a sample size of 20 completers (10 per sequence) was considered sufficient to achieve 90% power for demonstrating with > 85% level of proof that the highest dose was significantly better than placebo (assuming improvement of ≤ 25%). To account for a dropout rate of about 25%, 24 subjects were planned to be included.

## Results

### Study population

The study was conducted at five study sites in the UK and two study sites in The Netherlands, between February 2019 and September 2019.

**Fig. 2:**
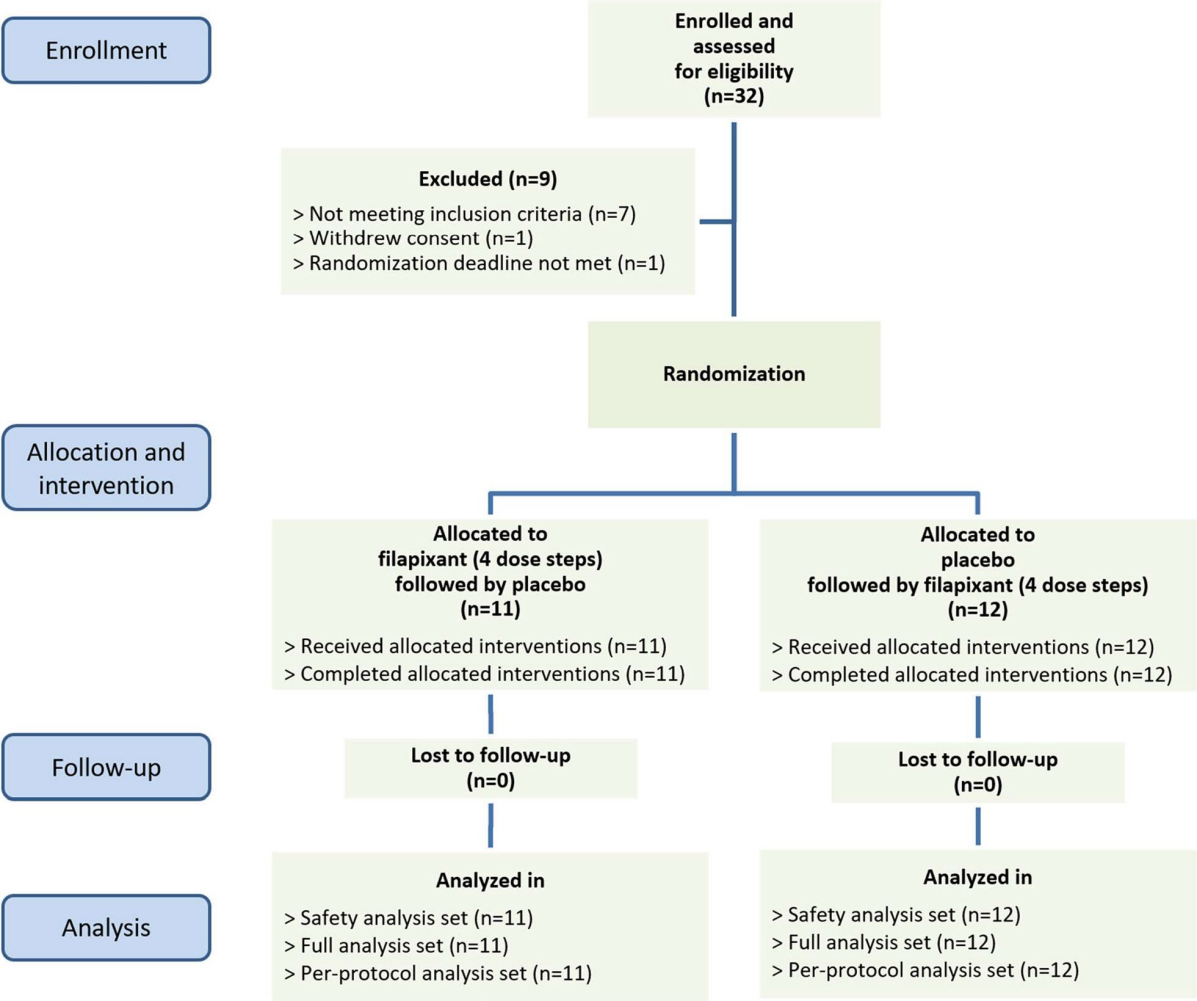
Patient disposition

In total, 23 patients with RCC were assigned to a treatment sequence, received their treatment in the assigned sequence, completed the study including the follow-up/end-of-study visit, and were included in all data analyses (Fig. 2). The baseline characteristics of these patients are shown in Table 1. The average baseline cough frequency was 28 coughs/h for the complete 24-h monitoring interval and 38 coughs/h while awake (geometric means). Use of concomitant medications was reported for 21 of 23 patients (91%), most often paracetamol [9 patients (39%)]. The (permitted) occasional use of opioids was reported for five patients in total—for one patient during both study periods and for four others only during the placebo period.

**Table 1:** Patient baseline characteristics

Age [years] (mean ± SD; range)	60.4 ± 9.1; 43–77
Age classification (< 65 years: ≥ 65 years) (N)	14 (16%): 9 (39%)
Sex (male: female ratio) (N)	5 (22%): 18 (78%)
Body weight [kg] (mean ± SD; range)	72.5 ± 11.9; 49.3–95.4
Body mass index [kg/m^2^] (mean ± SD; range)	26.8 ± 4.21; 19.6–34.3
Race	
Black or African American (N)	1 (4.3%)
White (N)	22 (95.7%)
Ethnicity	
Not Hispanic or Latino (N)	23 (100%)
Smoking history	
Never (N)	14 (60.9%)
Former (N)	9 (39.1%)
Other tobacco/cigar/pipe	
Former (N)	2 (8.7%)
Never (N)	21 (91.35)
Alcohol use	
Abstinent (N)	5 (21.75)
Light (N)	16 (69.65)
Moderate (N)	2 (8.75)
Duration of cough [years]^a^ (mean ± SD; range)	12.2 ± 10.02; 2–45
Baseline cough count (Period 1)	
24-h cough count [coughs/h] (geometric mean ± SD; range)	28.0 / 156; 0.7–513
Awake cough count [coughs/h] (geometric mean ± SD; range)	37.8 / 167; 1.0–745
Baseline LCQ scores (Period 1)	
LCQ total score (mean ± SD; range)	10.5 ± 3.16; 6.04–15.6
LCQ physical domain score (mean ± SD; range)	3.68 ± 1.10; 1.75–5.38
LCQ psychological domain score (mean ± SD; range)	3.67 ± 1.20; 1.43—5.43
LCQ social domain score (mean ± SD; range)	3.16 ± 1.24; 1.00—5.25

Percentages refer to the total number of study participants (N = 23).

LCQ, Leicester Cough Questionnaire (higher values indicate a better quality of life; domain scores can range from 1 to 7, total scores from 3 to 21); N, number of patients; SD, standard deviation. ^a^based on medical history entries with the high-level term ‘Coughing and associated symptoms’

### Efficacy

**Fig. 3:**
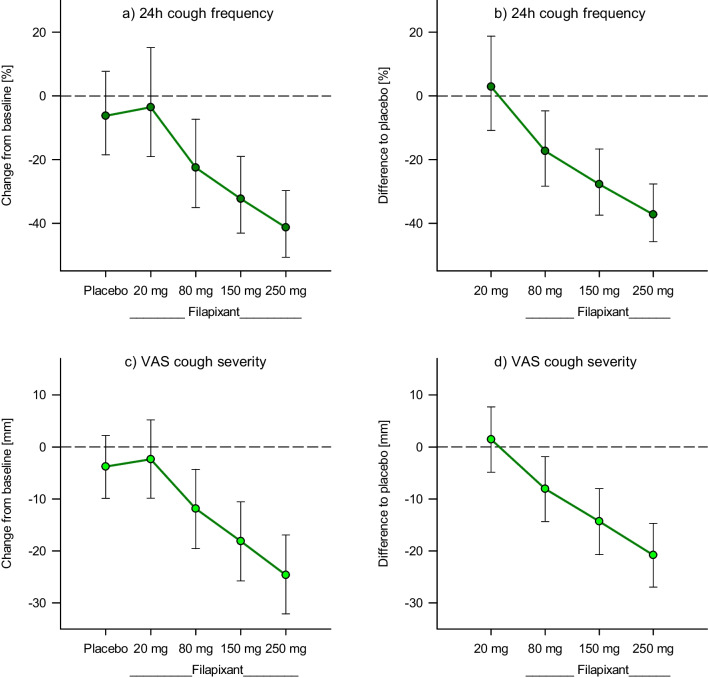
Cough frequency and severity (Day 4): Changes from baseline and differences to placebo (Bayesian analyses). Error bars represent 90% credible limits. Placebo data were pooled across the four placebo ‘dose’ steps. Lower VAS scores indicate less severe cough. Total number of patients = 23

After administration of filapixant at dosages ≥ 80 mg BID, the average *cough frequency* was reduced in a dose-dependent manner compared to baseline and relative to placebo (*P* < 0.05). This was the case both during the complete 24-h recording interval and while the patients were awake (Table 2a, b; Fig. 3a, b; Additional file 3: Table S1). The most pronounced reduction of cough frequency was observed with the 250 mg dose of filapixant for both the complete 24-h interval and awake hours, with a decrease of 41% and 43% from baseline, respectively. Relative to placebo, cough frequency reductions were 37% and 40% in the same time intervals.

**Table 2:** Cough frequency and severity, health-related quality-of-life: Changes from baseline and differences to placebo (Bayesian analyses)

Variable	Dose	Comparison with baseline				Comparison with placebo			
		%-change	Ratio to baseline [%]	90% credible limits [%]	*P*-value	%-difference	Ratio to placebo [%]	90% credible limits [%]	*P*-value
a) 24-h cough frequency [%]	Placebo	− 6.3	93.7	(81.5; 108)	0.222	N/A	N/A-	N/A	N/A
	20 mg	− 3.5	96.5	(80.9; 115)	0.365	3.0	103	(89.1; 119)	0.630
	80 mg	− 22.5	77.5	(65.0; 92.7)	0.010	− 17.3	82.7	(71.6; 95.3)	0.015
	150 mg	− 32.3	67.7	(56.9; 81.0)	< 0.001	− 27.7	72.3	(62.6; 83.4)	< 0.001
	250 mg	− 41.3	58.7	(49.3; 70.3)	< 0.001	− 37.2	62.8	(54.2; 72.4)	< 0.001
b) Awake cough frequency [%]	Placebo	− 4.9	95.1	(83.2; 108)	0.266	N/A	N/A	N/A	N/A
	20 mg	− 3.7	96.3	(81.4; 114)	0.356	1.0	101	(88.0; 117)	0.563
	80 mg	− 21.9	78.1	(65.7; 92.5)	0.0111	− 17.9	82.1	(71.4; 94.4)	0.012
	150 mg	− 33.8	66.2	(55.9; 78.6)	< 0.001	− 30.3	69.7	(60.4; 80.2)	< 0.001
	250 mg	− 43.1	56.9	(48.0; 67.6)	< 0.001	− 40.1	59.9	(51.9; 69.0)	< 0.001

		Difference treatment—baseline	90% credible limits	*P*-value	Difference active—placebo	90% credible limits	*P*-value
c) Cough severity (VAS) [mm]	Placebo	− 3.78	(− 9.87; 2.19)	0.148	N/A	N/A	N/A
	20 mg	− 2.36	(− 9.85; 5.20)	0.302	1.44	(− 4.84; 7.68)	0.649
	80 mg	− 11.8	(− 19.6; − 4.29)	0.007	− 8.05	(− 14.3; − 1.87)	0.017
	150 mg	− 18.1	(− 25.8; − 10.5)	< 0.001	− 14.3	(− 20.7; − 8.03)	< 0.001
	250 mg	− 24.6	(− 32.1; − 16.9)	< 0.001	− 20.8	(− 27.0; − 14.7)	< 0.001
d)							
LCQ total score	Placebo	1.07	(− 0.02; 2.15)	0.055	1.73	(0.58; 2.86)	0.008
	BAY	2.80	(1.71; 3.88)	< 0.001			
LCQ physical domain score	Placebo	0.36	(0.03; 0.68)	0.035	0.54	(0.15; 0.94)	0.014
	BAY	0.90	(0.57; 1.22)	< 0.001			
LCQ psychological domain score	Placebo	0.38	(− 0.00; 0.77)	0.050	0.46	(0.03; 0.89)	0.040
	BAY	0.85	(0.46; 1.23)	< 0.001			
LCQ social domain score	Placebo	0.31	(− 0.13; 0.75)	0.116	0.74	(0.36; 1.12)	0.002
	BAY	1.06	(0.63; 1.50)	< 0.001			

24-h cough counts were done on the 4th treatment day. Cough severity was assessed by the patient by means of a 100-mm VAS at the end of the 4th treatment day. The LCQ was completed before any treatments and at the end of each of the two treatment periods to assess the patient’s health-related quality of life. LCQ domain scores could range from 1 to 7, total scores from 3 to 21. Higher LCQ scores indicate a better quality of life. Total number of patients = 23

BAY, active treatment period; LCQ, Leicester Cough Questionnaire; N/A, not applicable. VAS, visual analog scale (lower values indicate less severe coughing)

The results of the responder analysis indicate that there was a predicted probability of 60% of experiencing a reduction in 24-h cough frequency by at least 30%, and a 45% predicted probability of a 50%-reduction in the same measure at the highest dose of filapixant (Table 3). Subgroup analyses by baseline cough count did not reveal any apparent trend of higher efficacy in patients with a higher cough count at baseline (Additional file 3: Table S2). Responder rates for both change thresholds and additionally for > 55% reduction^3^—a reduction observed by Schelfhout et al. [24] in patients who were classified as *very much improved/much improved* using the Patient Global Impression of Change scale—are provided in Additional file 3: Table S3. With filapixant 250 mg, seven of 23 patients (30%) achieved > 55% reduction (one patient (5%) with the time-matched placebo).

**Table 3:** Cough frequency: Responder analysis

Dose	> 30% reduction from baseline (complete 24-h interval)		> 50% reduction from baseline (complete 24-h interval)	
	Estimate [%]	90% credible limits	Estimate [%]	90% credible limits
Placebo	18.0	(11.3; 27.4)	4.79	(2.68; 8.40)
20 mg	20.3	(11.9; 32.5)	5.91	(3.05; 11.1)
80 mg	28.7	(13.9; 49.9)	10.9	(4.48; 24.2)
150 mg	40.6	(16.7; 70.0)	21.1	(6.96; 48.8)
250 mg	59.3	(21.3; 88.7)	44.8	(12.7; 81.9)

Total number of patients = 23

*Patient-reported cough severity* as assessed on a 100-mm VAS, was also reduced in a dose-dependent manner after 4-day administration of filapixant at dosages ≥ 80 mg BID (*P* < 0.05) (Table 2c; Fig. 3c, d). With the 250 mg dose, there was, on average, a decrease of 25 mm compared to baseline and of 21 mm compared to placebo. With filapixant 250 mg, eight of 23 patients (34.8%) achieved a reduction of > 30 mm in cough severity VAS scores, compared to 4 of 23 patients (17.4%) with the time-matched placebo (Additional file 3: Table S4).

**Fig. 4:**
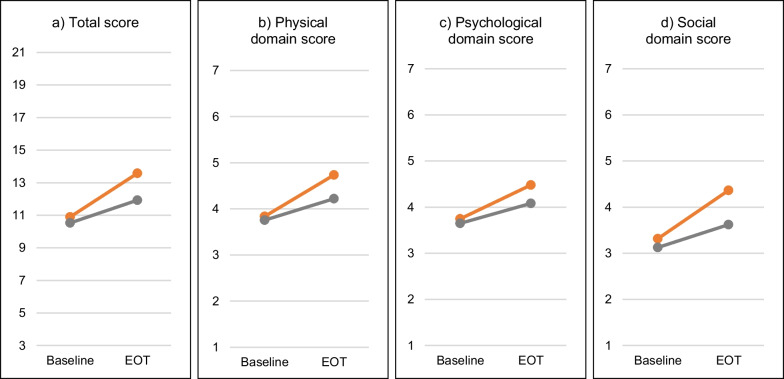
Leicester Cough Questionnaire: Mean total score and domain scores at baseline and at the end of treatment (EOT). Red lines and symbols: active treatment period; gray lines and symbols: placebo period. Higher values indicate a better quality of life. Total number of patients = 23

The *LCQ total scores* were markedly increased after administration of filapixant, indicating an overall improvement in the patients’ health-related quality of life (Fig. 4a). The mean change from baseline amounted to 2.80 points (*P* < 0.001), the difference to placebo was 1.73 points (*P* = 008) (Table 2d). Similar improvements in comparison to placebo and baseline were seen in the LCQ domain scores (Fig. 4b–d).

### Pharmacokinetics

The trough concentrations of filapixant determined on the morning of the 4th day of treatment with filapixant 20, 80, 150, and 250 mg BID increased in an approximately dose proportional manner from 14.7 µg/L [87.5%], 70.5 µg/L [42.1%], and 109 µg/L [156%] to 198 µg/L [210%] (geometric means and coefficients of variation). (For post-dose concentration–time curves, see Additional file 3: Figure S1).

### Safety and tolerability

Treatment-emergent AEs were reported for 22 of the 23 study participants (details in Additional file 3: Table S5). All AEs were mild or moderate; none of the AEs was serious or resulted in discontinuation of treatment. Overall, *dysgeusia* and *headache* were the most frequently reported AEs (MedDRA preferred terms) (8 patients [35%] each) followed by unspecified *taste disorders* (6 patients, 26%), and *nausea* (5 patients, 22%).

Taste-related AEs were reported mainly but not exclusively during treatment with filapixant (13 patients on filapixant; 3 patients on placebo). Qualitative changes in taste perception *(dysgeusia)* were described slightly more frequently than quantitative changes *(ageusia or hypogeusia)* (6 versus 4 patients receiving filapixant; 2 patients versus none receiving placebo). *Taste disorders* without further specification were reported by 5 patients receiving filapixant and by 1 patient receiving placebo.^4^ Most changes in taste perception were mild. Moderate taste changes were reported by 3 patients receiving 150 or 250 mg filapixant. All taste disorders were fully reversible, generally within hours or days. At the end-of-study/follow-up visit, all taste-related AEs had resolved.

In addition to the standard AE analysis with frequency counts based on the AE’s onset date only (Table 4), an additional “cumulative” analysis of all taste-related AEs (i.e., the preferred terms *hypogeusia, ageusia, dysgeusia,* and unspecified *taste disorder*) was carried out (Table 5). This analysis took not only the onset of an AE into consideration but also its duration. AEs starting at a lower dose level and persisting over two or more consecutive dose steps were counted at each dose level anew. This analysis showed that, overall, the frequency of taste-related AEs apparently increased with each increase of the filapixant dose.

**Table 4:** Taste-related adverse events (conventional analysis)

	Filapixant (N = 23)				Placebo (N = 23)			
Adverse event	20 mg	80 mg	150 mg	250 mg	(20 mg)	(80 mg)	(150 mg)	(250 mg)
(MedDRA preferred term)	N (%)	N (%)	N (%)	N (%)	N (%)	N (%)	N (%)	N (%)
Ageusia	0	1 (4.3%)	1 (4.3%)	0	0	0	0	0
Dysgeusia	1 (4.3%)	0	3 (13.0%)	5 (21.7%)	0	0	2 (8.7%)	0
Hypogeusia	0	1 (4.3%)	1 (4.3%)	1 (4.3%)	0	0	0	0
Taste disorder NOS	0	1 (4.3%)	4 (17.4%)	1 (4.3%)	1 (4.3%)	0	0	0
Total number of patients reporting such events	1 (4.3%)	2 (8.7%)	8 (34.8%)	6 (26.1%)	1 (4.3%)	0	2 (8.7%)	0

Percentages refer to the total number of patients (N = 23)

This table shows data only for the dose level at which the event first occurred, regardless of whether the event continued or recurred at subsequent dose levels. Adverse events were coded using MedDRA v22.0

**Table 5:** Taste-related adverse events (additional “cumulative” analysis)

Filapixant (N = 23)				Placebo (N = 23)			
20 mg	80 mg	150 mg	250 mg	(20 mg)	(80 mg)	(150 mg)	(250 mg)
N (%)	N (%)	N (%)	N (%)	N (%)	N (%)	N (%)	N (%)
1 (4.3%)	3 (13.0%)	10 (43.5%)	13 (56.5%)	2 (8.7%)	2 (8.7%)	4 (17.4%)	3 (13.0%)

The table provides the number of patients reporting taste-related adverse events for each dose step where such an event was present, irrespective of the time of its onset. The number of patients reporting taste-related AEs might decrease from one dose step to the next when taste disorders disappear in a patient. Taste-related adverse events were ageusia, dysgeusia, hypogeusia, and taste disorder without further specification (MedDRA v22.0 preferred terms)

No clinically relevant changes were noted in the safety laboratory parameters, vital signs (heart rate, blood pressure, and body temperature), and electrocardiogram assessments after treatment with filapixant.

## Discussion

Treatment with ascending doses of filapixant (80, 150, and 250 mg BID for 4 days each) was associated with a statistically significant, dose-dependent reduction in objective cough frequency and subjective cough severity, and an improvement in the patient-reported health-related quality of life in comparison to placebo. Consistent improvements of these endpoints were seen for dosages ≥ 80 mg BID with maximum effects on cough frequency and severity at the highest dosage, 250 mg filapixant BID—but without a clear plateau for either variable. The probability of experiencing a > 30% reduction in 24-h cough frequency—which can be regarded as a clinically meaningful cut-off point for a positive response according to Schelfhout et al. [24] – was estimated to be 60% at the highest dosage.

The absolute reduction in 24-h and awake cough frequency observed after 250 mg filapixant (approximately 40%) was similar to that observed after 750 mg eliapixant [13] or 50 mg gefapixant [11]. However, such comparisons across studies are generally problematic due to differences in treatment durations and placebo-adjustments.

The reduction in subjective cough severity VAS at the highest dose level (25 mm reduction from baseline) exceeded the threshold of ≥ 17 mm described in the literature as the minimal clinically important difference in patients with acute cough [22, 25], but it was slightly below the *clinically meaningful change threshold for clinical trials in chronic cough* of ≥ 30 mm recently presented by Martin Nguyen et al. [26].

The observed decrease in LCQ total scores, on the other hand, well exceeded the 1.3 points determined by Raj et al. as the minimal important difference for the LCQ total score [27]. Of note: The LCQ scores reflect the impact coughing had on the patient’s life during the preceding two weeks. In our study, a 4-days-on/3-days-off treatment schedule with ascending doses was used. Thus, the LCQ scores obtained after the last dose in the active treatment period might underestimate the therapeutic potential of filapixant. Whether a longer duration of treatment with filapixant (at a dosage of 250 mg BID) leads to more pronounced effects remains to be seen.

No safety issues were observed during the short therapeutic intervention and the tolerability of the drug was good at all dose levels with no AEs reported that were severe, serious, or led to discontinuation of treatment. However, the adverse event data collected in this study suggest that filapixant, like gefapixant, influences the perception of taste although the incidence rates of taste changes might have been overestimated in our “cumulative” analysis, which was intended as a “worst-case analysis”.

Overall, the number of patients with taste-related AEs (ageusia, hypogeusia, dysgeusia, and taste disorder without further specification) was much higher in our study than in the above-mentioned study with eliapixant [13] (conventional analysis: 9 of 40 patients, 23%, with dysgeusia; “cumulative” assessment: 21% with taste-related AEs at the highest dosage). The same applies to a study with sivopixant, another new selective P2X3 receptor antagonist [28] (2 of 31 patients, 6.5%, with mild taste disturbances). In contrast to the observations made in the above-mentioned study with eliapixant—the number of patients affected by such AEs increased substantially with the dose in our study. At the two lower dose levels of filapixant and the four placebo dose steps only between 1 and 4 patients of 23 patients (4–13%) reported taste-related AEs. At the 150 mg and the 250 mg dose levels, in contrast, 10 (44%) and 13 patients (57%), respectively, out of 23 patients reported such AEs. (Note: The incidence rates are considerably lower when the standard onset- and change-related procedure for AE documentation is used; Additional file 3: Table S5).

Incident rates of > 50% for taste-related AEs are not unexpected for unselective P2X3 antagonists, as such rates and also an association between dose and incidence rates of taste disturbances have been reported for gefapixant [11, 12]. However, filapixant has a higher in vitro selectivity for P2X3 than other receptor antagonists such as gefapixant, or even eliapixant, which is known as highly P2X3-selective. Therefore, only minimal taste-related side effects were expected. This suggests that other factors in addition to selectivity over P2X2/3 might explain the apparent discrepancy between clinically observed side effects and in vitro receptor selectivity. These additional factors might include the allosteric binding site of these compounds at the P2X3 receptor and differences in the pharmacokinetic characteristics of the drugs. One relevant difference between eliapixant and filapixant is the difference in the peak–trough fluctuation of plasma concentrations at steady state due to differences in half-lives. While there were little peak–trough fluctuations observed with eliapixant (ranging from 28.1% to 44.1%), fluctuations with filapixant are considerably higher (80 mg: 137%; 250 mg: 110%; data on file, Bayer). Based on preclinical data obtained with eliapixant [29], it is expected that with all P2X3 antagonists ~ 80% receptor occupancy needs to be maintained over the complete dosing interval to achieve full efficacy. Consequently, for compounds with large peak–trough fluctuations, the window between maximum plasma concentrations and antagonism of P2X2/3 will be diminished to a larger extent than for compounds with a small peak–trough fluctuations. In addition, it might be hypothesized that patients are more likely to become aware of rapid changes in P2X2/3 receptor occupancy (and the resulting changes in taste perception) as will happen with widely-fluctuating compounds in contrast to the slow changes seen with other agents. This would be in line with the lack of correlation between threshold measurements and self-reports of taste perception described by Cavazzana et al. [30], who point out that the awareness of sensory deficits might be reduced in older people because, with aging, such deficits usually develop slowly and gradually over time. Finally, additional experiments will have to be conducted to fully understand the differences in the incidence of taste-related AEs between filapixant and eliapixant, e.g., exact evaluations of the binding mode or comparisons of different dosing regimens (and pharmacokinetic profiles) in terms of their effects on taste perception.

Our study was designed as an exploratory study in a modest number of subjects and with very short treatment duration at each dose step. Thus, it has its obvious limitations. A larger study will have to follow to assess the long-term safety and tolerability of filapixant. For this proof-of-concept study, however, a 4-day treatment period was sufficient as previous studies with gefapixant [11, 12, 31] and eliapixant [13] have shown that the reduction of cough frequency observed after 4-day treatment is predictive of reduction to be observed after 12- or 24-week treatment.

The occasional, concomitant use of antitussives, in particular opioids, might be regarded as another problem of the study. A look at the data, however, shows that only one patient occasionally used opioids during the filapixant period (and also during the placebo period), while four patients occasionally used opioids during the placebo period. Thus, any impact on the study results in favor of filapixant seems unlikely. A third problem might be that the occurrence of taste disturbances might have led to a partial unmasking of the treatment allocation. Knowledge of the nature of the treatment received might have influenced the patient’s treatment response. Smith et al. have observed more substantial improvement in cough frequency in patients with taste disturbances than in patients without such disturbances [11]. A post-hoc subgroup analysis of our data, showed a similar tendency (Additional file 3: Table S6, S7), which however was not observed with eliapixant.

## Conclusions

Filapixant proved to be efficacious, safe, and—apart from the occurrence of taste disturbances, especially at higher dosages—well tolerated during the short therapeutic intervention.

## Supplementary Information

**Additional file 1.** Inclusion/exclusion criteria.

**Additional file 2.** Analytical methods.

**Additional file 3: Table S1.** 24-h cough monitoring: Cough count [1/hour]. **Table S2.** 24-h cough frequency: Subgroup analyses by baseline cough count (Bayesian mixed model). **Table S3.** 24-h cough frequency: Responder rates for different change thresholds. **Table S4.** VAS cough severity: Responder rates. **Table S5.** Treatment-emergent adverse events observed in > 5% of patients. **Table S6.** Cough frequency: Subgroup analyses by occurrence of taste-related adverse events (Bayesian mixed model). **Table S7.** Cough severity (VAS): Subgroup analyses by occurrence of taste-related adverse events (Bayesian mixed model). **Figure S1.** Plasma concentration–time curves for filapixant after multiple administrations.

**Additional file 4.** List of independent ethics committees consulted.

## Data Availability

The study protocol and the datasets generated and/or analyzed during the current study are not publicly available but are available from the corresponding author on reasonable request.

## Abbreviations

AE: Adverse events
BID: Twice daily
LCQ: Leicester Cough Questionnaire
RCC: Refractory chronic cough
VAS: Visual analog scale

## Footnotes

1 In everyday clinical practice, the same empirical treatment regimens are used for RCC and for unexplained chronic cough.

2 This study was the second part of a 2-part study with filapixant (BAY1902607). The first part of the study was a classical double-blind, randomized, placebo-controlled, phase 1 study in healthy male subjects (data on file, Bayer). Its objective was to investigate the safety, tolerability, pharmacokinetics, and pharmacodynamics of ascending multiple doses of filapixant (20, 80, and 250 mg twice daily over 2 weeks). The results of this first part will be reported in a separate publication. Part 2 was initiated only after careful review of the relevant safety and pharmacokinetic data obtained in Part 1. Otherwise, the two parts were independent of each other. The dose range studied in Part 2 corresponds to that of Part 1.

3 Post hoc analysis.

4 The following verbatim reports were coded into the term taste disorder without further specification: Altered taste sensation, changed taste, changed taste experience for water, taste disturbance(s), and taste disturbance overall.
