# Supplementary material for: The P2X3 receptor antagonist filapixant in patients with refractory chronic cough: a randomized controlled trial
Source: Respir Res. 2023 Apr 11;24:109. doi: 10.1186/s12931-023-02384-8 (PMC10088222; doi:10.1186/s12931-023-02384-8)
Supplement: Supplementary file 1 — Additional file 1. Inclusion/exclusion criteria. [file 12931_2023_2384_MOESM1_ESM.pdf]

C. Friedrich, K. Francke, S. S. Birring, J. W. K. van den Berg, P. A. Marsden, L. McGarvey, A. M. Turner, P. Wielders, I. Gashaw, S. Klein, A. Morice

The P2X3 receptor antagonist filapixant in patients with refractory chronic cough – a randomized trial

Respiratory Research 2023

Additional File 1

Inclusion/Exclusion Criteria

Inclusion criteria

Patients (of either sex) had to fulfill all of the following criteria before being included in the treatment period:

1. Signature of the informed consent form before any study-specific tests or procedures
2. Age: ≥18 years at the first screening visit
3. BMI: ≥18 kg/m^2 and ≤35 kg/m^2
4. Refractory chronic cough for at least 1 year that has been shown to be unresponsive to treatment of cough according to the 2006 British Thoracic Society guideline
5. Score of >40 mm on the Cough Severity 100-mm visual analog scale (VAS) at screening
6. *For male patients:*

   Male patients, who were sexually active and had not been surgically sterilized, had to agree to use two reliable and acceptable methods of contraception simultaneously (one method used by the study patient, one method used by the partner) during the study and for 90 days after receiving the study drug, and not to act as sperm donor for 90 days after dosing. Acceptable methods of contraception included for example: (a) condoms (male or female) with or without a spermicidal agent, (b) diaphragm or cervical cap with spermicide, (c) intrauterine device, (d) hormone-based contraception.

   For female patients:

   Confirmed post-menopausal woman (defined as exhibiting spontaneous amenorrhea for at least 12 months before screening or as exhibiting spontaneous amenorrhea for 6 months before screening with documented serum follicle-stimulating hormone [FSH] levels >40 mIU/mL) or

   woman without childbearing potential based on surgical treatment at least 6 weeks before screening, such as bilateral tubal ligation, bilateral oophorectomy with or without hysterectomy (documented by medical report verification) or

   woman of childbearing potential who agreed to use two reliable and acceptable methods of contraception simultaneously (one method used by the study patient and one method used by the partner) during the study and for at least 31 days (1 average menstrual cycle of 28 days plus approximately 5 half-lives of BAY 1902607) after the last dose. In addition, during the study and for at least 31 days after the last dose, women of childbearing potential were not allowed to donate oocytes. Acceptable methods of contraception included for example: (a) condoms (male or female) with or without a spermicidal agent, (b) diaphragm or cervical cap with spermicide, (c) intrauterine device, (d) hormone-based contraception.
7. Ability to understand and follow study-related instructions.

Exclusion criteria

Patients were excluded from the study if they displayed any of the following criteria:

Medical and surgical history

1. Forced expiratory volume in 1 second (FEV1) or forced vital capacity (FVC) of less than 60% of predicted normal, at screening
2. History of upper or lower respiratory tract infection or recent significant change in pulmonary status within the 4 weeks before screening
3. Severe renal impairment (estimated glomerular filtration rate [eGFR] <30 mL/min)
4. Moderate or severe liver impairment defined by clinically relevant deviations of laboratory liver function parameters from their respective reference ranges (especially alanine aminotransferase [ALT], aspartate aminotransferase [AST], bilirubin, serum albumin, international normalized ratio [INR]) or other clinically relevant findings
5. Severe cardiovascular diseases (e.g., history of myocardial infarction, unstable angina pectoris, thromboembolism)

Medication, drug use and special behavioral patterns

6. Current smoking habit or history of smoking within the 6 months before the screening visit
7. History of smoking (at any time) for more than 20 pack-years in total (20 cigarettes per pack)
8. Use of any systemic or topically active drug that might influence the pharmacokinetics of the study drug within the 14 days before first administration of study drug or during the study until the follow-up examination. [Note: This included repeated use of e.g., laxatives, loperamide, metoclopramide, any broad-spectrum antibiotic, or any use of CYP3A4 inducers or CYP3A4 inhibitors.]
9. Regular use of any systemic or topically active drug that modulated cough – such as acetylcholine esterase inhibitors, opioids, pregabalin, gabapentin, or any over the counter (OTC) antitussive agent - within the 14 days before first administration of study drug or during the study until the follow-up examination
10. History of concurrent malignancy or recurrence of malignancy within the 2 years before screening (this did not apply to enrolling subjects who had a malignancy <3 excised basal cell carcinomas)

Electrocardiogram, blood pressure, heart rate

11. Systolic blood pressure below 100 mmHg or above 160 mmHg (after at least 10 min of supine rest)
12. Diastolic blood pressure below 60 mmHg or above 100 mmHg (after at least 10 min of supine rest)
13. Heart rate below 50 bpm or above 95 bpm (after at least 10 min of supine rest)
14. Clinically significant abnormal electrocardiogram at screening (especially second- or third-degree atrioventricular block or hints or evidence for long QT syndrome) Laboratory examination
15. Clinically relevant deviations of the screened laboratory values from their respective reference ranges (especially persistent elevation of liver enzymes >2x upper limit of norm [ULN] for ALT and/or AST and/or >1.5x ULN for bilirubin)

Other

16. Current pregnancy or breast-feeding
17. Other severe, acute, or chronic medical or psychiatric condition or laboratory abnormality that might increase the risk associated with participation in the study or administration of the investigational product or might interfere with the interpretation of study results and, in the judgment of the investigator or the sponsor, would make the subject inappropriate for entry into this study
18. Previous (i.e., within the last 2 months or a longer and more appropriate time as determined by the investigator, e.g., approximately 5 half-lives of the previous investigational drug) or concomitant participation in another clinical study with investigational medicinal product(s)
19. Subject was in custody by order of an authority or a court of law
20. Criteria that in the opinion of the investigator precluded participation for scientific reasons, for reasons of compliance, or for reasons of the subject's safety
21. Previous assignment to treatment (i.e., randomization) during this study
22. Scheduled (elective) surgery or planned hospitalization during the period between signing the informed consent form and 6 weeks after the last administration of the study drug
23. Prolonged immobilization, major surgery, any surgery to the legs or major trauma unless complete remobilization has been achieved at least 4 weeks before the first screening examination
24. Close affiliation with the investigational site (e.g., a close relative of the investigator) or dependent person (e.g., employee/student of the investigational site)
25. Subject was an employee of the sponsor
26. Inability/unwillingness to comply with study restrictions
