# Supplementary material for: The P2X3 receptor antagonist filapixant in patients with refractory chronic cough: a randomized controlled trial
Source: Respir Res. 2023 Apr 11;24:109. doi: 10.1186/s12931-023-02384-8 (PMC10088222; doi:10.1186/s12931-023-02384-8)
Supplement: Supplementary file 2 — Additional file 2. Analytical methods. [file 12931_2023_2384_MOESM2_ESM.pdf]

C. Friedrich, K. Francke, S. S. Birring, J. W. K. van den Berg, P. A. Marsden, L. McGarvey, A. M. Turner, P. Wielders, I. Gashaw, S. Klein, A. Morice

**The P2X3 receptor antagonist filapixant in patients with refractory chronic cough – a randomized trial**

*Respiratory Research 2023*

# Additional File 2

# Analytical Methods

Quantitative analysis of filapixant in plasma was performed using a fully validated method. The validation of the method and the analysis of the study samples were performed in compliance with the pertinent FDA and EMA guidelines.

Briefly, filapixant (free base) was determined in plasma after protein precipitation with acetonitrile/ammonium acetate buffer, 2 nM, pH3; (6/1; v/v) containing an internal standard followed by filtration and subsequent evaporation to dryness with nitrogen at about 50 °C. Afterwards, the residues were reconstituted with ammonium acetate buffer, 2 nM, pH3/acetonitrile (8/2; v/v), followed by separation employing high-pressure LC-MS/MS.

The calibration range of the procedure was 0.100 µg/L (LLOQ) to 100 µg/L (ULOQ). The mean inter-assay accuracy of back-calculated concentrations in calibrators (except LLOQ) ranged between 97.9% and 103% and precision was ≤4.67%. Accuracy and precision at the lowest calibrator (LLOQ) were equal to 102% and 4.69%, respectively. Quality control samples in the concentration range from 0.300 to 80.0 µg/L were determined with an accuracy of 97.3% to 102% and a precision of 3.80% to 5.84%. All samples were stored at or below -15 °C and analyzed within 173 days after sampling. The stability data indicated that the analyte was stable for this time period.
