# Supplementary material for: The P2X3 receptor antagonist filapixant in patients with refractory chronic cough: a randomized controlled trial
Source: Respir Res. 2023 Apr 11;24:109. doi: 10.1186/s12931-023-02384-8 (PMC10088222; doi:10.1186/s12931-023-02384-8)
Supplement: Supplementary file 3 — Additional file 3: Table S1. 24-h cough monitoring: Cough count [1/hour]. Table S2. 24-h cough frequency: Subgroup analyses by baseline cough count (Bayesian mixed model). Table S3. 24-h cough frequency: Responder rates for different change thresholds. Table S4. VAS cough severity: Responder rates. Table S5. Treatment-emergent adverse events observed in > 5% of patients. Table S6. Cough frequency: Subgroup analyses by occurrence of taste-related adverse events (Bayesian mixed model). Table S7. Cough severity (VAS): Subgroup analyses by occurrence of taste-related adverse events (Bayesian mixed model). Figure S1. Plasma concentration–time curves for filapixant after multiple administrations. [file 12931_2023_2384_MOESM3_ESM.pdf]

C. Friedrich, K. Francke, S. S. Birring, J. W. K. van den Berg, P. A. Marsden, L. McGarvey, A. M. Turner, P. Wielders, I. Gashaw, S. Klein, A. Morice

The P2X3 receptor antagonist filapixant in patients with refractory chronic cough – a randomized trial

Respiratory Research 2023

Additional File 3

Supplemental tables and figures

Table of supplemental tables

Table S 1: 24-h cough monitoring: Cough count [1/hour] 2
Table S 2: 24-h cough frequency: Subgroup analyses by baseline cough count (Bayesian mixed model) 2
Table S 3: 24-h cough frequency: Responder rates for different change thresholds 3
Table S 4: VAS cough severity: Responder rates 3
Table S 5: Treatment-emergent adverse events observed in >5% of patients 4
Table S 6: Cough frequency: Subgroup analyses by occurrence of taste-related adverse events (Bayesian mixed model) 5
Table S 7: Cough severity (VAS): Subgroup analyses by occurrence of taste-related adverse events (Bayesian mixed model) 6

Table of supplemental figures

Figure S 1: Plasma concentration-time curves for filapixant (BAY1902607) after multiple administrations 7

Table S 1: 24-h cough monitoring: Cough count [1/hour]

	Complete 24-h interval		Awake phases	
	**Geom. Mean / %CV**	**(Range)**	**Geom. Mean / %CV**	**(Range)**
**Active drug period**				
Baseline	27.2 / 181	(0.6-581)	36.0 / 194	(0.8-766)
20 mg filapixant	26.1 / 193	(0.4-521)	34.5 / 214	(0.6-740)
80 mg filapixant	21.0 / 213	(0.3-645)	28.0 / 219	(0.5-875)
150 mg filapixant	18.4 / 207	(0.9-713)	23.8 / 216	(1.1-931)
250 mg filapixant	16.0 / 233	(0.5-600)	20.5 / 242	(0.7-832)
**Placebo period**				
Baseline	24.6 / 151	(0.7-513)	32.5 / 159	(1.0-745)
PLC4	23.3 / 181	(0.4-671)	31.0 / 181	(0.6-794)
PLC11	24.4 / 156	(1.5-806)	32.4 / 153	(2.1-1020)
PLC18	22.6 / 177	(0.4-720) [a]	31.6 / 177	(0.6- 903) [a]
PLC25	21.4 / 171	(0.3- 686)	27.8 / 181	(0.5-993)

%CV, coefficient of variation [%]; PLC4, placebo given from Day 0 to Day 3; PLC11, placebo given from Day 7 to Day 10; PLC18; placebo given from Day 14 to Day 17; PLC25, placebo given from Day 21 to Day 24. [a] Number of evaluable patients = 22. Total number of patients = 23.

Table S 2: 24-h cough frequency: Subgroup analyses by baseline cough count (Bayesian mixed model)

			Comparison with baseline				Comparison with placebo			
	**Baseline cough count**	**Dose**	***%-change***	**Ratio to baseline [%]**	**90% credible limits [%]**	**P-value**	***%-difference***	**Ratio to placebo [%]**	**90% credible limits [%]**	**P-value**
A	**Cough frequency ≥5/h** (N=22)	Placebo	-6.3	93.7	(80.7; 109)	0.235		--	--	--
		20 mg	-3.1	96.9	(80.9; 117)	0.389	3.9	104	(89.6; 120)	0.666
		80 mg	-22.3	77.7	(64.7; 93.3)	0.013	-17.0	83.0	(71.8; 95.8)	0.017
		150 mg	-33.7	66.3	(55.2; 80.0)	0.000	-29.1	70.9	(61.0; 81.9)	<0.001
		250 mg	-41.9	58.1	(48.3; 69.9)	<0.001	-37.9	62.1	(53.5; 71.8)	<0.001
B	**Cough frequency ≥15/h** (N=18)	Placebo	-8.1	91.9	(77.7; 108)	0.190	--	--	--	--
		20 mg	-3.7	96.3	(78.7; 119)	0.381	5.1	105	(89.0; 124)	0.695
		80 mg	-28.0	72.0	(58.7; 88.3)	0.006	-21.5	78.5	(66.6; 92.4)	0.008
		150 mg	-36.3	63.7	(52.0; 78.3)	0.000	-30.5	69.5	(58.7; 81.8)	<0.001
		250 mg	-45.9	54.1	(44.0; 66.6)	<0.001	-41.1	58.9	(50.0; 69.6)	<0.001
C	**Cough frequency ≥30/h** (N=11)	Placebo	-7.2	92.8	(70.8; 118)	0.291	--	--	--	--
		20 mg	0.7	101	(73.7; 137)	0.516	9.1	109	(86.2; 139)	0.736
		80 mg	-20.4	79.6	(58.4; 108)	0.104	-13.9	86.1	(67.9; 109)	0.147
		150 mg	-32.1	67.9	(50.0; 92.2)	0.020	-26.4	73.7	(57.7; 93.3)	0.016
		250 mg	-38.4	61.6	(45.5; 83.8)	0.007	-33.2	66.8	(52.6; 84.7)	0.004

Total number of patients = 23.

Table S 3: 24-h cough frequency: Responder rates for different change thresholds

	>30% reduction from baseline		>50% reduction from baseline		>55% reduction from baseline [a]		
	N of responders	N of non-responders	N of responders	N of non-responders	N of esponders	N of non-responders	Total
**Active drug period**							
20 mg filapixant	2 (8.7%)	21 (91.3%)	0 (0.0%)	23 (100.0%)	0 (0 %)	23 (100 %)	23 (100.0%)
80 mg filapixant	9 (39.1%)	14 (60.9%)	4 (17.4%)	19 (82.6%)	2 (8.7 %)	21 (91.3 %)	23 (100.0%)
150 mg filapixant	11 (47.8%)	12 (52.2%)	8 (34.8%)	15 (65.2%)	6 (26.1 %)	17 (73.9 %)	23 (100.0%)
250 mg filapixant	12 (52.2%)	11 (47.8%)	8 (34.8%)	15 (65.2%)	7 (30.4 %)	16 (69.6 %)	23 (100.0%)
**Placebo period**							
PLC4	2 (8.7%)	20 (87.0%)	0	22 (95.7%)	0 (0 %)	22 (100 %)	22 (95.7%)
PLC11	2 (8.7%)	20 (87.0%)	1 (4.3%)	21 (91.3%)	1 (4.5 %)	21 (95.5 %)	22 (95.7%)
PLC18	5 (21.7%)	16 (69.6%)	0	21 (91.3%)	0 (0 %)	21 (100 %)	21 (91.3%)
PLC25	6 (26.1%)	16 (69.6%)	2 (8.7%)	20 (87.0%)	1 (4.5 %)	21 (95.5 %)	22 (95.7%)

N = number; PLC4, placebo given from Day 0 to Day 3; PLC11, placebo given from Day 7 to Day 10; PLC18; placebo given from Day 14 to Day 17; PLC25, placebo given from Day 21 to Day 24. Total number of patients = 23.
[a] post hoc analysis

Table S 4: VAS cough severity: Responder rates

	>30 mm reduction from baseline [a]		
	Number of responders	Number of non-responders	Total
**Active drug period**			
20 mg filapixant	3 (13.0%)	20 (87.0%)	23 (100.0%)
80 mg filapixant	8 (34.8%)	15 (65.2%)	23 (100.0%)
150 mg filapixant	7 (30.4%)	16 (69.6%)	23 (100.0%)
250 mg filapixant	8 (34.8%)	15 (65.2%)	23 (100.0%)
**Placebo period**			
PLC4	1 (4.3%)	22 (95.7%)	23 (100.0%)
PLC11	2 (8.7%)	21 (91.3%)	23 (100.0%)
PLC18	2 (9.1%)	20 (90.9%)	22 (95.7%)
PLC25	4 (17.4%)	19 (82.6%)	23 (100.0%)

PLC4, placebo given from Day 0 to Day 3; PLC11, placebo given from Day 7 to Day 10; PLC18; placebo given from Day 14 to Day 17; PLC25, placebo given from Day 21 to Day 24. Total number of patients = 23.
[a] post hoc analysis

Table S 5: Treatment-emergent adverse events observed in >5% of patients

MedDRA v22.0<br>Primary system organ class<br>Preferred term	20 mg BAY	80 mg BAY	150 mg BAY	250 mg BAY	PLC4	PLC11	PLC18	PLC25	Total
	N (%)	N (%)	N (%)	N (%)	N (%)	N (%)	N (%)	N (%)	N (%)
Patients with any AE	*11 (47.8%)*	*9 (39.1%)*	*11 (47.8%)*	*13 (56.5%)*	*10 (43.5%)*	*9 (39.1%)*	*7 (30.4%)*	*8 (34.8%)*	*22 (95.7%)*
**Gastrointestinal disorders**	3 (13.0%)	5 (21.7%)	5 (21.7%)	5 (21.7%)	2 (8.7%)	5 (21.7%)	2 (8.7%)	1 (4.3%)	16 (69.6%)
Abdominal pain upper	0	1 (4.3%)	0	0	0	0	0	1 (4.3%)	2 (8.7%)
Diarrhoea	1 (4.3%)	0	0	1 (4.3%)	0	0	1 (4.3%)	0	3 (13.0%)
Dry mouth	0	0	1 (4.3%)	1 (4.3%)	0	0	1 (4.3%)	0	3 (13.0%)
Glossodynia	0	1 (4.3%)	0	1 (4.3%)	0	0	0	0	2 (8.7%)
Nausea	2 (8.7%)	0	1 (4.3%)	0	2 (8.7%)	2 (8.7%)	0	0	5 (21.7%)
Paraesthesia oral	0	1 (4.3%)	1 (4.3%)	1 (4.3%)	0	0	0	0	2 (8.7%)
Vomiting	0	1 (4.3%)	2 (8.7%)	0	0	1 (4.3%)	0	1 (4.3%)	3 (13.0%)
**General disorders and administration site conditions**	2 (8.7%)	1 (4.3%)	1 (4.3%)	0	0	0	1 (4.3%)	1 (4.3%)	4 (17.4%)
Fatigue	0	1 (4.3%)	1 (4.3%)	0	0	0	0	0	2 (8.7%)
**Injury, poisoning and procedural complications**	1 (4.3%)	1 (4.3%)	0	0	0	0	0	0	2 (8.7%)
Contusion	1 (4.3%)	1 (4.3%)	0	0	0	0	0	0	2 (8.7%)
Investigations	0	0	0	0	1 (4.3%)	1 (4.3%)	1 (4.3%)	0	2 (8.7%)
**Musculoskeletal and connective tissue disorders**	0	1 (4.3%)	0	1 (4.3%)	1 (4.3%)	0	0	3 (13.0%)	5 (21.7%)
Arthralgia	0	0	0	0	0	0	0	2 (8.7%)	2 (8.7%)
**Nervous system disorders**	4 (17.4%)	4 (17.4%)	8 (34.8%)	9 (39.1%)	5 (21.7%)	3 (13.0%)	4 (17.4%)	2 (8.7%)	18 (78.3%)
Ageusia	0	1 (4.3%)	1 (4.3%)	0	0	0	0	0	2 (8.7%)
Dizziness	0	1 (4.3%)	1 (4.3%)	0	1 (4.3%)	1 (4.3%)	0	0	3 (13.0%)
Dysgeusia	1 (4.3%)	0	3 (13.0%)	5 (21.7%)	0	0	2 (8.7%)	0	8 (34.8%)
Headache	1 (4.3%)	1 (4.3%)	2 (8.7%)	2 (8.7%)	4 (17.4%)	2 (8.7%)	2 (8.7%)	2 (8.7%)	8 (34.8%)
Lethargy	1 (4.3%)	0	0	0	0	1 (4.3%)	0	0	2 (8.7%)
Hypogeusia	0	1 (4.3%)	1 (4.3%)	1 (4.3%)	0	0	0	0	3 (13.0%)
Taste disorder NOS	0	1 (4.3%)	4 (17.4%)	1 (4.3%)	1 (4.3%)	0	0	0	6 (26.1%)
**Respiratory, thoracic and mediastinal disorders**	0	0	2 (8.7%)	1 (4.3%)	0	0	1 (4.3%)	2 (8.7%)	3 (13.0%)
Cough	0	0	2 (8.7%)	1 (4.3%)	0		1 (4.3%)	1 (4.3%)	3 (13.0%)
**Skin and subcutaneous tissue disorders**	0	0	1 (4.3%)	1 (4.3%)	1 (4.3%)	1 (4.3%)	0	1 (4.3%)	4 (17.4%)
Pruritus	0	0	1 (4.3%)	0	1 (4.3%)	0	0	0	2 (8.7%)
Vascular disorders	1 (4.3%)	2 (8.7%)	0	0	2 (8.7%)	0	0	0	4 (17.4%)
Haematoma	1 (4.3%)	1 (4.3%)	0	0	2 (8.7%)	0	0	0	3 (13.0%)

Percentages refer to the total number of patients (N=23). Taste-related AEs are highlighted with grey shading.
AE, adverse event; BAY, filapixant; N, numbers of patients; NOS, no other specification; PLC4, placebo given from Day 0 to Day 3; PLC11, placebo given from Day 7 to Day 10; PLC18; placebo given from Day 14 to Day 17; PLC25, placebo given from Day 21 to Day 24. Total number of patients = 23.

Table S 6: Cough frequency: Subgroup analyses by occurrence of taste-related adverse events (Bayesian mixed model)

			Comparison with baseline		Comparison with placebo	
		**Dose**	**Ratio to baseline**	**90% credible limits**	**Ratio to placebo**	**90% credible limits**
A	**With any taste-related AE(s)** (N13)	Placebo	0.90	(0.77; 1.05)	--	--
		20 mg	1.00	(0.82; 1.22)	1.11	(0.93; 1.33)
		80 mg	0.68	(0.56; 0.83)	0.75	(0.63; 0.90)
		150 mg	0.50	(0.41; 0.62)	0.56	(0.46; 0.67)
		250 mg	0.40	(0.32; 0.48)	0.44	(0.37; 0.53)
B	**Without any taste-related AE(s)** (N=10)	Placebo	1.07	(0.85; 1.34)	--	---
		20 mg	0.92	(0.71; 1.20)	0.86	(0.73; 1.01)
		80 mg	0.92	(0.71; 1.20)	0.86	(0.73; 1.01)
		150 mg	0.95	(0.73; 1.24)	0.89	(0.76; 1.04)
		250 mg	0.91	(0.70; 1.19)	0.85	(0.72; 0.99)
C	**With taste-related AE(s) with 250 mg BAY** (N=13)	Placebo	0.89	(0.75; 1.03)	--	--
		20 mg	1.00	(0.82; 1.25)	1.14	(0.93; 1.39)
		80 mg	0.66	(0.53; 0.81)	0.74	(0.61; 0.90)
		150 mg	0.48	(0.39; 0.59)	0.54	(0.45; 0.66)
		250 mg	0.37	(0.30; 0.45)	0.41	(0.34; 0.50)
D	**Without taste-related AE(s) with 250 mg BAY** (N=10)	Placebo	1.09	(0.92; 1.27)	--	--
		20 mg	0.92	(0.76; 1.14)	0.85	(0.73; 1.00)
		80 mg	0.89	(0.73; 1.10)	0.82	(0.70; 0.96)
		150 mg	0.90	(0.74; 1.10)	0.83	(0.71; 0.97)
		250 mg	0.86	(0.70; 1.05)	0.79	(0.68; 0.93)
E	**With taste-related AE(s) with 150 mg BAY** (N=10)	Placebo	0.83	(0.69; 0.99)	--	--
		20 mg	1.01	(0.79; 1.29)	1.22	(0.99; 1.52)
		80 mg	0.66	(0.52; 0.84)	0.80	(0.65; 0.99)
		150 mg	0.53	(0.42; 0.68)	0.64	(0.52; 0.80)
		250 mg	0.41	(0.32; 0.52)	0.49	(0.40; 0.61)
F	**Without taste-related AE(s) with 150 mg BAY** (N=13)	Placebo	1.08	(0.95; 1.23)	--	--
		20 mg	0.93	(0.78; 1.09)	0.86	(0.74; 0.98)
		80 mg	0.89	(0.75; 1.06)	0.83	(0.72; 0.95)
		150 mg	0.80	(0.67; 0.94)	0.74	(0.64; 0.85)
		250 mg	0.76	(0.64; 0.90)	0.70	(0.61; 0.81)
G	**With taste-related AE(s) with 80 mg BAY** (N=3)	Placebo	0.70	(0.02; 24.11)	--	--
		20 mg	1.03	(0.04; 38.98)	1.53	(0.78; 3.21)
		80 mg	0.77	(0.03; 28.39)	1.14	(0.58; 2.43)
		150 mg	0.44	(0.02; 15.84)	0.65	(0.33; 1.38)
		250 mg	0.42	(0.02; 15.65)	0.63	(0.32; 1.32)
H	**Without taste-related AE(S) with 80 mg BAY** (N=20)	Placebo	0.97	(0.83; 1.14)	--	--
		20 mg	0.98	(0.80; 1.19)	1.00	(0.86; 1.17)
		80 mg	0.79	(0.65; 0.96)	0.81	(0.70; 0.95)
		150 mg	0.70	(0.57; 0.85)	0.72	(0.61; 0.84)
		250 mg	0.59	(0.49; 0.72)	0.61	(0.52; 0.71)

Total number of patients = 23.

Table S 7: Cough severity (VAS): Subgroup analyses by occurrence of taste-related adverse events (Bayesian mixed model)

		Dose	Comparison with baseline: Change from baseline [mm]	Comparison with baseline: 90% credible limits [mm]	Comparison with placebo: Difference to placebo [mm]	Comparison with placebo: 90% credible limits [mm]
A	**With any taste-related AE(s)** (N=13)	Placebo	-5.96	(-13.06; 1.16)	--	--
		20 mg	-4.43	(-14.32; 5.00)	1.48	(-7.68; 10.54)
		80 mg	-16.89	(-26.77; -7.18)	-10.99	(-19.89; -1.85)
		150 mg	-29.24	(-39.10; -19.46)	-23.34	(-32.35; -14.23)
		250 mg	-37.92	(-47.94; -28.07)	-32.00	(-41.04; -22.93)
B	**Without any taste-related AE(s)** (N=10)	Placebo	0.07	(-11.09; 10.70)	--	--
		20 mg	1.33	(-10.73; 13.69)	1.25	(-5.70; 8.44)
		80 mg	-4.47	(-16.48; 7.62)	-4.47	(-11.53; 2.61)
		150 mg	-0.67	(-12.64; 11.27)	-0.75	(-7.87; 6.13)
		250 mg	-4.54	(-16.48; 7.79)	-4.50	(-11.41; 2.39)
C	**With taste-related AE(s) with 250 mg BAY** (N=13)	Placebo	-2.03	(-9.62; 5.33)		
		20 mg	-4.25	(-14.42; 5.48)	-2.17	(-11.56; 7.08)
		80 mg	-15.37	(-25.35; -5.33)	-13.34	(-22.64; -3.78)
		150 mg	-28.83	(-38.75; -18.90)	-26.73	(-36.05; -17.37)
		250 mg	-35.69	(-45.90; -25.76)	-33.64	(-43.04; -24.66)
D	**Without taste-related AE(s) with 250 mg BAY** (N=10)	Placebo	-2.83	(-14.63; 8.75)	--	--
		20 mg	0.20	(-12.77; 13.70)	3.05	(-4.61; 10.85)
		80 mg	-7.39	(-20.33; 5.48)	-4.58	(-12.16; 2.98)
		150 mg	-4.06	(-17.24; 8.88)	-1.23	(-8.80; 6.28)
		250 mg	-9.87	(-23.09; 3.09)	-7.02	(-14.70; 0.53)
E	**With taste-related AE(s) with 150 mg BAY** (N=10)	Placebo	0.65	(-7.91; 9.38)	--	--
		20 mg	-1.79	(-13.87; 9.46)	-2.58	(-13.98; 8.42)
		80 mg	-14.86	(-26.65; -3.14)	-15.62	(-26.77; -4.26)
		150 mg	-26.98	(-38.63; -15.23)	-27.66	(-38.68; -16.50)
		250 mg	-34.47	(-46.31; -22.94)	-35.22	(-46.53; -24.19)
F	**Without taste-related AE(s) with 150 mg BAY** (N=13)	Placebo	-6.12	(-16.06; 3.55)	--	--
		20 mg	-2.47	(-13.68; 8.57)	3.61	(-3.14; 10.62)
		80 mg	-9.48	(-20.68; 1.52)	-3.29	(-10.23; 3.59)
		150 mg	-11.19	(-22.34; 0.00)	-5.00	(-11.92; 1.87)
		250 mg	-16.85	(-28.08; -5.65)	-10.64	(-17.54; -3.84)
G	**With taste-related AE(s) with 80 mg BAY** (N=3)	Placebo	-3.21	(-74.07; 75.42)	--	--
		20 mg	-5.31	(-76.79; 74.16)	-2.14	(-15.37; 11.05)
		80 mg	-32.57	(-104.0; 45.17)	-29.42	(-42.54; -16.03)
		150 mg	-51.87	(-123.9; 25.91)	-48.58	(-61.65; -35.36)
		250 mg	-61.58	(-132.4; 17.23)	-58.44	(-71.55; -45.04)
H	**Without taste-related AE(S) with 80 mg BAY** (N=20)	Placebo	-3.75	(-10.63; 2.75)	--	--
		20 mg	-1.69	(-9.92; 6.61)	2.15	(-4.12; 8.41)
		80 mg	-7.98	(-16.23; 0.06)	-4.18	(-10.52; 2.06)
		150 mg	-12.68	(-20.98; -4.68)	-8.97	(-15.27; -2.67)
		250 mg	-18.66	(-26.73; -10.51)	-14.79	(-21.12; -8.55)

Total number of patients = 23.

Figure S 1: Plasma concentration-time curves for filapixant (BAY1902607) after multiple administrations

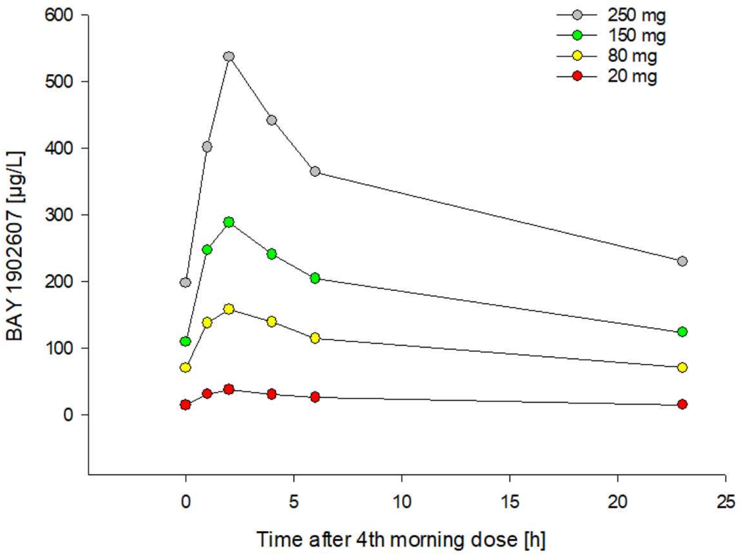

Total number of patients = 23.
