# Supplementary material for: The P2X3 receptor antagonist filapixant in patients with refractory chronic cough: a randomized controlled trial
Source: Respir Res. 2023 Apr 11;24:109. doi: 10.1186/s12931-023-02384-8 (PMC10088222; doi:10.1186/s12931-023-02384-8)
Supplement: Supplementary file 4 — Additional file 4. List of independent ethics committees consulted. [file 12931_2023_2384_MOESM4_ESM.pdf]

C. Friedrich, K. Francke, S. S. Birring, J. W. K. van den Berg, P. A. Marsden, L. McGarvey, A. M. Turner, P. Wielders, I. Gashaw, S. Klein, A. Morice

The P2X3 receptor antagonist filapixant in patients with refractory chronic cough – a randomized trial

Respiratory Research 2023

Additional File 4

List of independent ethics committees consulted

For study centers in the United Kingdom:

North West - Greater Manchester Central REC
HRA Centre Manchester
3rd Floor
Barlow House
M1 3DZ Manchester
United Kingdom

For study centers in The Netherlands:

Isala
METC, Isala
Gebouw Mondriaan, kamer 0.25
Postbus 10400
8000 GK ZWOLLE
The Netherlands
